# Perceptions on healthy eating, physical activity and lifestyle advice: opportunities for adapting lifestyle interventions to individuals with low socioeconomic status

**DOI:** 10.1186/1471-2458-14-1036

**Published:** 2014-10-04

**Authors:** Andrea J Bukman, Dorit Teuscher, Edith J M Feskens, Marleen A van Baak, Agnes Meershoek, Reint Jan Renes

**Affiliations:** Division of Human Nutrition, Wageningen University, P.O Box 8129, 6700 EV Wageningen, The Netherlands; Department of Human Biology, NUTRIM School for Nutrition, Toxicology and Metabolism, Maastricht University Medical Centre+, P.O Box 616, 6200 MD Maastricht, The Netherlands; Department of Health, Ethics and Society, CAPHRI, Maastricht University Medical Centre+, P.O Box 616, 6200 MD Maastricht, The Netherlands; Division of Strategic Communication, Wageningen University, P.O box 8130, 6700 EW Wageningen, The Netherlands

**Keywords:** Socioeconomic status, Perceptions on lifestyle, Physical activity, Healthy eating, Focus groups

## Abstract

**Background:**

Individuals with low socioeconomic status (SES) are generally less well reached through lifestyle interventions than individuals with higher SES. The aim of this study was to identify opportunities for adapting lifestyle interventions in such a way that they are more appealing for individuals with low SES. To this end, the study provides insight into perspectives of groups with different socioeconomic positions regarding their current eating and physical activity behaviour; triggers for lifestyle change; and ways to support lifestyle change.

**Methods:**

Data were gathered in semi-structured focus group interviews among low SES (four groups) and high SES (five groups) adults. The group size varied between four and nine participants. The main themes discussed were perceptions and experiences of healthy eating, physical activity and lifestyle advice. Interviews were transcribed verbatim and a thematic approach was used to analyse the data.

**Results:**

In general, three key topics were identified, namely: current lifestyle is logical for participants given their personal situation; lifestyle change is prompted by feedback from their body; and support for lifestyle change should include individually tailored advice and could profit from involving others. The perceptions of the low SES participants were generally comparable to the perceptions shared by the high SES participants. Some perceptions were, however, especially shared in the low SES groups. Low SES participants indicated that their current eating behaviour was sometimes affected by cost concerns. They seemed to be especially motivated to change their lifestyle when they experienced health complaints, but were rather hesitant to change their lifestyle for preventive purposes. Regarding support for lifestyle change, low SES participants preferred to receive advice in a group rather than on their own. For physical activities, groups should preferably consist of persons of the same age, gender or physical condition.

**Conclusions:**

To motivate individuals with low SES to change their lifestyle, it may be useful to (visually) raise their awareness of their current weight or health status. Lifestyle interventions targeting individuals with low SES should take possible cost concerns into account and should harness the supportive effect of (peer) groups.

**Electronic supplementary material:**

The online version of this article (doi:10.1186/1471-2458-14-1036) contains supplementary material, which is available to authorized users.

## Background

Persons with low socioeconomic status (SES) are more likely to have poorer health and a shorter life expectancy than persons with higher SES [1]. These differences can partly be explained by a less favourable lifestyle [2]. In general, persons with low SES are less likely to eat healthily [3, 4] and are less likely to be physically active during leisure time [5–7]. This makes the low SES group an important target group for lifestyle interventions, given that these interventions are found to be an effective way to improve lifestyle and consequently reduce the risk of chronic diseases [8–11].

Although the effects of such lifestyle interventions are promising, individuals with low SES are less likely to perceive the need for lifestyle advice [12] and participate less often in these lifestyle interventions than individuals with high SES [13, 14]. Moreover, individuals with low SES who initially participate in these interventions might be more likely to drop out than individuals with high SES [11, 15]. Apparently, different approaches are necessary to successfully reach individuals with low SES for lifestyle interventions. For this reason, the focus of this study is on identifying possibilities for making an intervention potentially more applicable to individuals with low SES.

Tailoring a lifestyle intervention to the targeted individuals’ needs is a promising strategy for developing effective lifestyle interventions [16]. Tailoring can be effected in various ways, such as by mentioning the name of the targeted individual in a message or by including personal feedback on an individual’s behaviour [17]. However, to improve the effectiveness of lifestyle interventions, it is important not only to tailor the message, but also to choose the appropriate source, setting and channel for the health communication [18, 19]. A meta-analysis of interventions that promoted physical activity showed that the mode of delivery is important when socioeconomically disadvantaged women are being targeted. Interventions that included a group element in their intervention achieved better results than interventions with individual or community-based delivery [20].

A tailored intervention should suit the targeted individuals’ needs, and it should be realised that these needs may differ from those standardly perceived by health professionals. Several researchers have argued that future health promotion activities should pay more attention to the perceptions of the target group, instead of following the standard principles of health promotion and science-based understandings of a healthy lifestyle [21, 22]. Consumers’ definition of a healthy diet, for example, appears to be broader than the scientific definition that focuses on food composition and health outcomes [23].

Likewise, it should be realised that there is a friction between the health-oriented view of researchers and health promoters and the complexity of participants’ everyday life [24]. The perceived difficulty of fitting intervention activities into participants’ personal life can be an important barrier to engaging in health promoting programmes [25]. In addition, an accumulation of personal problems can hinder participants from engaging in lifestyle change [26]. Therefore, more attention should be paid to the complexity of participants’ everyday life [24]. To make lifestyle interventions better suited to participants’ day-to-day practices, it is important to get insight into the target group’s perceptions regarding a healthy lifestyle and lifestyle advice.

People’s perceptions are to some extent related to socioeconomic position. One study showed socioeconomic differences in the perceived relevance of various food topics and the need for information on these topics [27]. It observed, for example, that high SES participants were more interested in receiving information about food composition than low SES participants. In line with this, another study showed different barriers to physical activity among individuals with different socioeconomic status [28]. It suggested, for example, that, especially among low SES groups, health-promoting activities should take account of neighbourhood safety and negative early life experiences with physical activity. This indicates that different barriers or interests need to be taken into account when lifestyle interventions targeting individuals with either high or low SES are being created or adapted.

The aim of the current study was to identify opportunities for adapting lifestyle interventions in such a way as to make them more appealing and accessible to individuals with low socioeconomic status. To this end, the study provided insights into people’s perspectives regarding healthy eating, physical activity and lifestyle advice, with special attention on the following questions:

1. How do low SES participants explain their own eating behaviour and physical activity pattern?
2. What can trigger low SES participants to change their lifestyle?
3. How do low SES participants believe that they can be supported in lifestyle change?

This study addressed perspectives among groups with different socioeconomic positions in order to understand what perspectives exist in general and what perspectives may exist in particular among individuals with low SES that should be taken into account in developing a lifestyle intervention.

## Methods

### Study design

Nine focus group interviews were conducted in two Dutch provinces, namely, Gelderland and Limburg. In each province, the interviews were carried out among two low SES groups and two or three high SES groups (men and women separately). The reason for separating the focus groups by gender was to create more homogeneous groups, since the flow of an interview was expected to be smoother in more homogenous groups compared to mixed groups [29]. The study was not, however, intended to examine differences between genders. Beforehand, it was expected that four groups per socioeconomic group would be enough to reach saturation [30]. As a result of convenience sampling, an additional ninth group volunteered to participate in the study. The number of participants per group varied between four and nine, with a total of 56 participants. All participants were born in the Netherlands. The average age of the participants was 57.1 ± 9.0 years (range = 39–75 years). The participants’ characteristics are presented in Table 1. The study was approved by the medical ethics committee of Maastricht University. All participants gave written informed consent and received a gift voucher of 10 euros for participating in the focus group interviews.

**Table 1:** **Characteristics of focus group interview participants (mean ± sd or n (%))**

	Participants in low SES groups (n = 26)	Participants in high SES groups (n = 30)
**Age (years)**	60.3 ± 7.7	54.4 ± 9.2
**BMI (kg/m** ^**2**^ **)**	27.8 ± 3.8	24.7 ± 3.6
Education level*:		
Low	16 (61.5)	0 (0.0)
Middle	9 (34.6)	2 (6.7)
High	1 (3.8)	28 (93.3)
**Employment status:**		
Paid job/own company	9 (34.6)	26 (86.7)
Househusband/housewife	5 (19.2)	1 (3.3)
Retired	9 (34.6)	3 (10.0)
Disabled	3 (11.5)	0 (0.0)
**Marital status:**		
Married	17 (65.4)	22 (73.3)
Unmarried	3 (11.5)	7 (23.3)
Divorced	2 (7.7)	1 (3.3)
Widow (er)	4 (15.4)	0 (0.0)
**Household situation:**		
Alone	7 (26.9)	6 (20.0)
Together with partner	16 (61.5)	13 (43.3)
Together with partner and child (ren)	3 (11.5)	11 (36.7)

*Participants who had no education, or had primary school or lower secondary education were classified as low education level. High education level was defined as having completed at least a bachelor’s degree.

### Procedure

The focus group interviews were held with pre-existing groups, specifically groups of persons who already met regularly (for example in a community centre or at an association). Individuals were asked in person to participate in a focus group interview by the researchers or via a member or contact person of the group. In order to reach groups with low SES, persons in community centres or associations in more deprived areas were approached. Higher socioeconomic groups were recruited by contacting members of associations in which normally persons with a higher socioeconomic position are involved (e.g. university setting or rotary club). The time and location of the interviews were determined by the participants themselves, and were often the time and location at which the group regularly met. Several days before the interview, participants received written information about the procedure. The interviews lasted approximately 1.5 to 2.5 hours. Following the interview, a short questionnaire was used to determine age, country of birth, marital status, household situation, employment status, highest completed education, height and weight. Two researchers (AJB and DT) were in charge of recruitment. The researcher who recruited the participants also moderated the focus group interview, and the other researcher observed. The interviews were conducted between May 2011 and November 2011.

### Interview guide

This study addressed different perspectives and experiences about healthy eating, physical activity and lifestyle advice. A semi-structured interview guide was developed around these topics based on literature relating to qualitative studies and theory on behaviour change [31]. The interview guide contained open-ended questions about participants’ daily eating practice; experiences and perceptions regarding barriers, enablers and social influences for healthy eating and physical activity; and earlier experiences and future needs relating to lifestyle advice (see Additional file 1).

### Data analysis

The interviews were audiotaped and transcribed verbatim. All transcripts were individually read by two researchers (AJB and DT) and frequently emerging themes were identified. These themes were discussed to create one coding scheme. Data were coded with NVivo 9 (QSR international Pty Ltd, Doncaster, Victoria, Australia). One transcript was coded by both researchers independently and discussed together afterwards. Only a few discrepancies were observed, which were discussed by the two researchers to reach consensus about the coding process. Because of these discrepancies, the researchers chose to slightly adapt the coding scheme by combining themes and renaming themes, to make it more suitable for the coding of the transcripts. The remaining transcripts were finally coded by the first author of this article. Thereafter, the researcher (AJB) went through the themes to identify key topics relating to healthy eating, physical activity and lifestyle advice in order to find out what is important for participants in current lifestyle, lifestyle change and support for lifestyle change. Within the topics, special attention was paid to the perceptions of low SES participants compared to those of high SES participants, to see whether some arguments might have been exclusively mentioned by individuals with either low or high SES. Quotes illustrative of the identified topics were selected.

## Results

**Figure 1:**
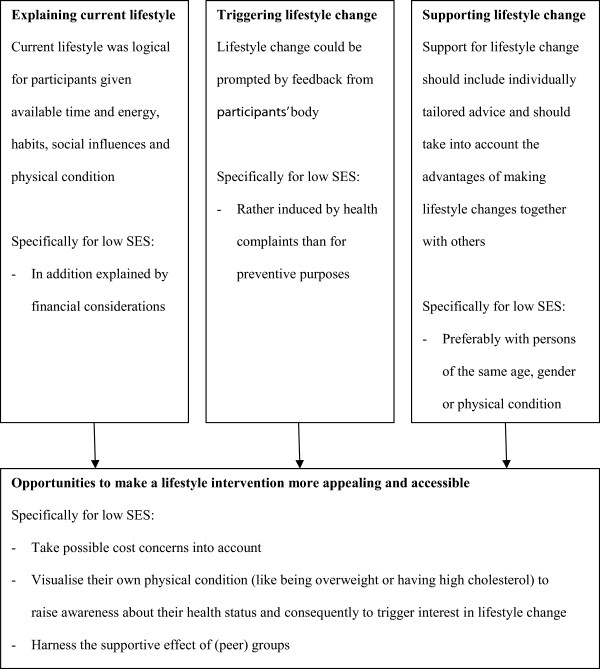
**Overview study results and identified opportunities for lifestyle interventions targeting individuals with low SES.**

Three key topics relating to eating behaviour, physical activity and lifestyle advice were identified, namely: current lifestyle is logical for participants given their personal situation; lifestyle change is prompted by feedback from their body; and support for lifestyle change should include individually tailored advice and should take into account the advantages of making lifestyle changes together with others. The perceptions of the low SES participants were in general comparable to the perceptions shared by the high SES participants. Some perceptions were, however, especially shared in the low SES groups. The perceptions regarding the three key topics are summarised in Figure 1 and described in more detail below.

### Current lifestyle is logical for participants given their personal situation

Frequently, participants indicated that their current lifestyle – healthy or not – worked for them. Physical activity and eating behaviour were explained in both SES groups as logical with regard to their: available time and energy, habits, social influences and physical condition. Especially in the case of some low SES participants, eating behaviour was in addition explained by financial considerations. Some participants stated that they simply did not have the motivation to eat more healthily or to be more physically active.

#### Time and energy

For those participants motivated to live healthily, having enough time and energy was an important requirement for having a healthy diet. Participants indicated that preparing *a healthy meal* could take more time and effort.

*“I think it is a disadvantage, or maybe not really a disadvantage, but that it [eating healthily] takes more time sometimes. Or you have to prepare it properly, that you peel the potatoes earlier, or something like that.” (Low SES woman, 49 years old)*

Participants also indicated that a lack of time or a lack of energy after a long day’s work could make it sometimes difficult to be *physically active*. Participants perceived that they had to divide their time and energy. Physical activities, for example, had to compete with other activities.

*“I should do it [exercise] more often, but sometimes the motivation is lacking, and the time. At home the laundry is waiting for me. And then you have to make choices: Will I do the laundry or am I going to exercise? Do I choose to take care of my mother, or am I going to do other things? Choices.” (Low SES woman, 44 years old)*

Participants also mentioned that, if they planned their eating behaviour and physical activities, it became easier to do it.

*“What I did notice, what does help – not that I always do it, but I do have those periods that I do – is when you plan it. You make up some recipes for a few days and you do the groceries for that.” (High SES woman, 48 years old)*

*“If you, for example, like me, go for a walk with a friend on Tuesday evening, and she knows that, you know, I will be there on Tuesday evening at seven o’clock.” (High SES woman, 60 years old)*

#### Habits

Some participants indicated that it was easy for them to live healthily because that was how they grew up or it was what they were used to doing.

*“I was raised to eat quite healthily. But if you are not used to that, I think it can be difficult.” (Low SES man, 69 years old)*

However, other participants indicated that it was difficult for them to live healthily because they were used to the unhealthy behaviour. Likewise, some participants indicated that healthy behaviours should become habitual, but that, at the moment, these healthy behaviours were rather an exception than a rule for them.

*“My husband and I often say it; we go walking before we go to bed or around half past nine in the evening. But it should become a habit. It is now rather an exception.” (Low SES woman, 61 years old)*

#### Financial cost

Low SES groups in particular discussed the influence of cost on their shopping and *eating behaviour*. They mentioned that they did their grocery shopping at cheap supermarkets and indicated that special offers influenced their food choice. They furthermore considered higher financial cost as a disadvantage of eating healthily.

*“As I understand from you, money is a disadvantage for healthy eating. Are there any other disadvantages?” (Interviewer)*

*“I think money is the most important factor.” (Low SES woman, 64 years old)*

*“That is the most important.” (Low SES woman, 56 years old)*

*“You can’t take whatever you want. You have to pay attention to the price. With everything. We first had two incomes, but we don’t have my income anymore. (…) Then you really need to pay attention to the things you buy.” (Low SES woman, 62 years old)*

The high SES groups that discussed the higher cost of healthy foods put this into perspective by saying that a healthy diet might be cheaper in the long run, taking into account the total lifestyle and the long-term health costs.

*“It is about your lifestyle as a whole and then I think that eating healthily does not have to be more expensive.” (High SES man, 60 years old)*

*“It could be that it [eating healthily] is even cheaper.” (High SES man, 47 years old)*

*“In the end, I am convinced of that. If you take into account the medical cost in the long term, etcetera.” (High SES man, 60 years old)*

#### Social influences

All groups indicated that the social environment made it sometimes difficult to *eat healthily*. Enjoying an alcoholic beverage or an unhealthy snack was often associated with sociability. At a party or in a social setting, participants sometimes found it difficult to resist unhealthy foods.

*“When you are at a reception or whatever – that happens once, twice or three times a month or something – then I think: ‘Oh, no’. I find that difficult, when you want to eat healthily, but you get stuck in a snack situation.” (High SES woman, 53 years old)*

Another reason why it could be difficult for participants to say no was because they did not want to disappoint the hostess.

*“Then you do not want to displease someone, or they have bought a lot of food. Then you think I will eat a little. That is how it goes.” (Low SES woman, 44 years old)*

At home also, it sometimes became difficult for participants to eat healthily because family members bought unhealthy products or because family members did not want to join them in eating healthy alternatives. At the same time, participants could be stimulated by their family members to eat healthily by improving their eating behaviour together or by following the good example of family members who already ate healthily.

*“When the persons in your surrounding eat more healthily, you are going to do that more easily as well. My wife thinks it important to eat healthily, my daughter as well. But especially my wife influences me, because she is always around. I think your surroundings play a decisive role.” (High SES man, 61 years old)*

Some participants indicated that they ate more healthily by adapting their own eating pattern to the needs or wishes of family members, or that family members adapted their eating patterns to what the participants needed.

*“I have to pay attention because of the diabetes as well. So, my husband does that automatically as well. He gets the same [food]. I am not going to prepare two types of vegetables and two types of potatoes, or whatever. I make all the same. But he doesn’t mind.” (Low SES women, 56 years old)*

Social influences were also noticeable in participants’ perceptions regarding *physical activity*. For some participants, physical activity was a social occasion, associated with the opportunity to meet new people. Being part of a group made it easier for participants to go to exercise sessions, because they felt obligated to go even if they had other things to do or felt no motivation at that moment.

*“Then you have that appointment. And then you won’t cancel it that easily. Then you really first need to have a good excuse.” (Low SES woman, 49 years old)*

Family members, especially the partner and children, could also motivate participants to exercise by saying they should be physically active or by joining them. Some participants indicated that their family members could also demotivate them, for example by reminding them of other things that should be done (first). Such competing activities, like household activities or family duties, could inhibit participants from being physically active.

*“You are getting older, you have kids, and you do not have any time anymore to exercise because you are busy with the kids and so on.” (Low SES man, 54 years old)*

#### Physical condition

Some participants stated that their physical condition made it difficult or impossible for them to be *physically active*.

*“That your body sometimes can’t do it [being physically active], because of certain health complaints.” (High SES woman, 51 years old)*

*“When I was 15 [years old], I started working at a building site, so my body is just not functioning anymore. It’s finished. Done.” (Low SES man, 62 years old)*

However, at the same time, as illustrated in the next section, someone’s physical condition could be a motivation to engage in a healthy lifestyle.

### Lifestyle change is prompted by feedback from their body

Participants relied strongly on the feedback that their own body gave them. Both low and high SES groups mentioned the negative health consequences of an *unhealthy diet* or a lack of *physical activity*. However, more than the high SES participants, the low SES participants stated that they first needed to get a signal from their own body before they would change their lifestyle.

*“As long as I feel healthy and I don’t suffer from anything, I eat whatever I want.” (Low SES man, 58 years old)*

However, some participants mentioned that it might be too late if they were to wait for a signal before improving their lifestyle. Like many high SES participants, some low SES participants stated that a healthy lifestyle was necessary to prevent overweight and health complaints.

*“But it is also for preventive purposes. To prevent all kind of things. When you eat fatty, you can get cardiovascular complaints.” (Low SES man, 54 years old)*

Several participants mentioned that they had already experienced some health complaints and stated that these health complaints were the trigger to change their lifestyle.

*“I have suffered three heart attacks. That’s why I take a little bit of care of what I eat.” (Low SES man, 54 years old)*

*“I have been in the hospital once, because of a heart attack. And then I have been reminded of some things. That is why I have changed my lifestyle.” (High SES man, 61 years old)*

Lifestyle change was also prompted by less extreme feedback from participants’ bodies, such as a simple change in weight:

*“What I did notice was that I weighed 106 kilograms at a certain point. I stood naked on my wife’s weighing scale. One hundred and six kilogrammes naked, then I scratched my head and started thinking: ‘how did it happen’? So, normally when I came home and was watching TV, then I always ate something before I went to bed. And now I consciously stopped doing that and I weigh 102 kilograms again.” (Low SES man, 58 years old)*

In the case of lifestyle advice also, several participants from both SES groups believed that their own body could tell them what was healthy for them and saw themselves as the most reliable source of information.

*“But your body will indicate it, what you can or can’t eat. Because when I eat more sauce than normally, I notice it immediately.” (Low SES woman, 62 years old)*

When participants discussed the possibility of receiving support for lifestyle change from health professionals, they indicated once more that it was person-specific support that was needed. As illustrated in the next section, participants therefore considered it important for health professionals to take a participant’s personal situation into account.

### Support for lifestyle change should include individually tailored advice and could profit from involving others

Participants made suggestions about how they could be supported to make lifestyle changes. They required tailored lifestyle advice and discussed the influence of involving significant others. In low SES groups in particular, the advantage of making lifestyle changes together with comparable others was mentioned.

Although some participants were keen to receive support for lifestyle change, others indicated that they were not interested. Some participants mentioned that they already lived healthily and therefore did not need advice. Others indicated that they already knew what was healthy or already received enough advice. Some men considered themselves too old to receive lifestyle advice.

*“If I was 20, I would say: ‘Yes I do need advice’. But not anymore at this time.” (Low SES man, 70 years old)*

Furthermore, as with lifestyle change, participants often felt that there needed to be something wrong with their weight or health before they would visit health professionals for lifestyle advice.

*“You often just don’t do it without a reason. You don’t just go to someone like that [nutritionist], there must be a reason.” (Low SES woman, 44 years old)*

#### Tailored lifestyle advice

Those participants who were interested in receiving advice mentioned that it was person-specific whether something was good for one. Therefore, they would like to receive tailored *nutrition* advice, preferably based on knowledge about how their own body works. Some high SES participants suggested that such individually tailored information could be given on the basis of the results of health checks.

*“You can give some general advice – like that is good and that is not good – but not personal advice. Then you first need at least maybe blood and urine tests and whatever more.” (High SES woman, 72 years old)*

In the case of *physical activity* guidance also, interested participants mentioned that the person giving the advice should understand the personal situation and physical condition of the participant, so that the advice could be tailored to the individual situation. Some low SES participants in addition mentioned that they wanted to get advice specifically for their age.

*“You become older. You become stiffer. Tying your shoelaces, that kind of things, all those movements become more difficult. I would like to get more specific physical activity advice about that” (Low SES man, 65 years old)*

#### Making lifestyle changes together

A change in lifestyle might be more easily accomplished together with others. Support for lifestyle change could make use of that by involving significant others. Some low SES participants in particular indicated that they would like to receive *nutrition* advice in a group. They explained that, in a group, members could stimulate one another by interchanging ideas and experiences and by social control.

*“In a group, you can accomplish more. At least, you will have more motivation. If I look into your eyes and I say: ‘I did not eat any potatoes this week’, you can’t check it. (…) But he lives next to me, and then he can say ‘I have seen you sitting at the table, with potatoes’.” (Low SES man, 58 years old)*

In contrast, high SES participants frequently indicated that they preferred to receive nutrition advice individually. They found that advice on an individual level could become more personally relevant or more specific, whereas on a group level it would often remain very general.

*“In a group, you get the more general [information], what you already know.” (High SES women, 60 years old)*

With regard to *physical activity*, participants from both SES groups indicated that they preferred to be physically active in a group rather than on their own. Participants found it more enjoyable to do physical activities with others. Additionally, being part of a group could stimulate them because others in the group would expect them to show up.

*“You don’t cancel it that easily. You made your appointment.” (High SES woman, 58 years old)*

The low SES participants in particular mentioned that it would be stimulating to exercise together with persons of the same age, gender, physical activity level or health complaints. One perceived advantage was that they could exercise on the same intensity level.

*“My daughter regularly exercises a few times a week. But I don’t think I will go together with my daughter, because I can’t keep up with her. (…) I can’t keep up the pace and then I would think ‘Sorry, I won’t join you’. If you are in a group with persons of the same age, then you have about the same tempo. (…) I would appreciate that.” (Low SES woman, 64 years old)*

Another advantage with respect to being physically active with comparable others was that participants expected to be better understood by other participants.

*“When you’re going to exercise with persons with the same illness, it is easier. (…) If you say that you have to take a break, you feel less awkward.” (Low SES woman, 56 years old)*

The support of similar peer groups could apparently help to create a safe and accessible setting for facilitating lifestyle change among these low SES participants.

## Discussion

This study addressed perceptions of low and high SES groups regarding healthy eating, physical activity and lifestyle advice and provided insight into the variety of perceptions – which exist either in general or more specifically among low SES groups – that should be taken into account when a lifestyle intervention is being adapted for individuals with low SES. The results showed three striking aspects regarding current lifestyle, lifestyle change and support for lifestyle change. In general, participants described their current lifestyle – healthy or not – as logical for them given their personal situation in terms of their available time and energy, habits, social influences and physical condition. In order to change their lifestyle, participants first had to be prompted by feedback given by their own body. With regard to supporting this lifestyle change, participants indicated that it was important to tailor lifestyle advice towards their personal situation. The perceptions of the low SES participants were in general quite comparable to the perceptions shared by the high SES participants. However, some perceptions were especially shared among the low SES groups. Low SES participants indicated that their current eating behaviour was sometimes affected by cost concerns. They seemed to be especially motivated to change their lifestyle when they experienced health complaints, but were rather hesitant to change their lifestyle for preventive purposes. Furthermore, they preferred to receive lifestyle advice in groups and to be physically active in a group of persons of the same gender, age or physical condition.

The low SES groups in this study seemed to be more affected by cost in their current lifestyle than the high SES groups. Financial cost was more often mentioned by the low SES groups and more intensively mentioned as making a real difference in their food choices. When high SES participants brought up the topic of cost, they put it more into perspective, for example by mentioning that cost concerns could be an issue for other persons. Financial cost is a recurring theme in research among low SES groups. Cost is often cited as an influence or barrier in food choices among low SES groups [32–35]. For physical activity however, cost concerns were hardly mentioned as a barrier by our groups. This is in accordance with another qualitative study, which showed that financial cost was not perceived as a key barrier for physical activity in any of their SES groups [28]. Some other studies, however, did show that financial cost could be a barrier to starting or continuing physical activity among individuals with low SES [36, 37]. More generally, losing weight is more often experienced as expensive by less educated persons compared to more highly educated persons [38]. Apparently, cost could be an issue for individuals with low SES with respect to lifestyle (change), and therefore participants’ possible cost concerns should be taken into account in lifestyle interventions.

The observation that our low SES participants were mostly not prevention oriented is in line with other studies that observed that individuals with lower SES are less likely to think about ways to stay healthy [39], are less likely to control their weight [38] and health status [40] and are in general less interested in screening activities [41–43]. Our participants indicated that they expected their body to warn them when something was wrong with their health. Several participants mentioned that they had already experienced health complaints and cited their health complaints as the trigger to engage in healthy behaviour. Likewise, Van der Waerden and colleagues observed that an increased severity of complaints is associated with a greater willingness to participate in, and keep following, prevention programmes [44]. Apparently, some persons first have to experience health complaints or changes in their physical condition before they become motivated to change their behaviour. Therefore, it can be a challenge to motivate these persons to participate in preventive activities. A possible solution could be to use individuals’ own physical condition (like being overweight or having high cholesterol) or the signs that their own body gives as the trigger to make individuals aware of their own current health status and the possible benefits of lifestyle change.

To support this lifestyle change, lifestyle interventions for low SES persons could profit from the supportive effect of (peer) groups. Low SES participants in particular preferred dietary advice and physical activities together with others. Involving friends, families and peers in order to create social support is a strategy that is often suggested in order to promote healthy lifestyles among low SES groups [37, 45, 46]. A review of lifestyle interventions stimulating physical activity among women with low SES showed that lifestyle interventions with a group component were more effective [20]. Being part of a group can help to make these persons feel more accountable and therefore more motivated [47]. Our low SES participants especially preferred to be physically active together with persons of the same age, gender or health complaints. This finding may be bound up with the on-average higher age and BMI of our low SES participants compared to our high SES participants. However, that seems rather a speculative statement given that none of our high SES participants – of whom some were also relatively older and overweight – expressed this preference. Another study among women in deprived neighbourhoods also observed that being physically active together with participants with similar health conditions could be encouraging [36]. Lifestyle change is easier to accomplish together with (the social support of) others, and including a group component in lifestyle interventions might be extremely important for targeting low SES individuals.

Some methodological choices should be taken into consideration in relation to interpreting the results. Although the focus group interviews gave rich and detailed data on the variety of perceptions that may exist among groups with different socioeconomic status, this method is not suitable for arriving at firm conclusions about actual differences between socioeconomic groups. In general, the study does give us a better understanding of the variety of perceptions that exists among groups with different socioeconomic status, which – regardless of whether these perceptions are more common among individuals with either low or high SES – should be considered in developing a lifestyle intervention. Moreover, we observed some perspectives that were exclusively shared by our low SES participants and supported by the existing literature; this finding may further help to make a lifestyle intervention more appealing and accessible to individuals with low SES.

In this research, participants were recruited via pre-existing groups. Participants were already acting in a social group, and therefore it could be that our groups were more focused on social support and group activities. Individuals that are not acting in a social group might have other perceptions regarding lifestyle advice in groups. However, the fact that our participants were acting in a social group would not completely explain why our low SES participants preferred lifestyle advice and physical activities in groups, whereas our high SES groups – also pre-existing groups – were less willing to receive nutrition advice in groups. Likewise, another study demonstrated with the help of survey research and individual interviews that being physically active together with others is an enabler or pre-requisite for individuals with low SES to participate in physical activities [37].

Our study gives valuable information on how individuals in the target group find that a healthy lifestyle fits into their life; what motivates them to participate in lifestyle change; and how this change can be facilitated, according to them. As already mentioned, these perceptions of the target group can differ from the perceptions of health professionals. Therefore, it is interesting to get insight into how the ideas of the target group match with the experiences of health professionals and whether participants’ suggestions for supporting lifestyle change actually suit the practicalities. A next step is to study how the revealed insights for adapting lifestyle interventions aimed at individuals with low SES can be realised in a real-life situation.

## Conclusions

This study gave important insights into perceptions relating to healthy eating, physical activity and lifestyle advice of individuals with different socioeconomic positions, and reveals some promising opportunities to adapt lifestyle interventions especially for individuals with low SES. To motivate individuals with low SES to participate in a lifestyle intervention, it may be useful to visualise their own physical condition (like being overweight or having high cholesterol) to raise their awareness about their health status and consequently to trigger interest in lifestyle change. Lifestyle interventions targeting individuals with low SES should take possible cost concerns into account and should harness the supportive effect of peer groups.

## Electronic supplementary material

Additional file 1: **Interview questions.** (DOCX 31 KB)
