# Supplementary material for: Perceptions on healthy eating, physical activity and lifestyle advice: opportunities for adapting lifestyle interventions to individuals with low socioeconomic status
Source: BMC Public Health. 2014 Oct 4;14:1036. doi: 10.1186/1471-2458-14-1036 (PMC4210550; doi:10.1186/1471-2458-14-1036)
Supplement: Supplementary file 1 — Additional file 1: Interview questions. (DOCX 31 KB) [file 12889_2014_7172_MOESM1_ESM.docx]

| **Questions with which to start the conversation:** | **Ask why! Continue with the following questions:** |
| --- | --- |
| ***Introduce yourself*** |  |
| What did you have for dinner last night? | - Do you often eat that type of meal? |
| Who usually prepares dinner? |  |
| Who usually does the grocery shopping? |  |
| Where do you usually buy your groceries? |  |
| ***Nutrition*** |  |
| If I say food, what do you think about? | - How important is food for you? |
| Do you think that you eat healthily? | - What is healthy? - What is unhealthy? |
| Is it important for you to eat healthily? | - What do you see as **advantage(s)** of eating healthily? - What do you see as **disadvantage(s)** of eating healthily? |
| Would you like to eat more healthily? | - What would you like to change about your eating behaviour? Have you ever tried that? How did you try and how did you experience that? - What makes it **difficult** for you to eat healthily? - What makes it **easier** for you to eat healthily? |
| By whom are you **supported** to eat healthily? | - Which people **discourage** you from eating healthily (disapprove of you eating healthily)? - How do you experience that? |
| Do you talk with others about what you eat? About what? | - From whom do you take advice? How do you experience that advice? - What are for you trustworthy sources regarding information about healthy nutrition? |
| Would you like to receive advice about healthy eating? | - From whom? - About what topic would you like to receive advice? |
| Where and when would you like to receive advice about healthy eating? | - Individually or in a group? |
| ***Physical activity*** |  |
| How did you get here? | - Is that the way you usually do that? |
| Do you like to be physically active? | - What do you see as **advantage(s)** of being physically active? - What do you see as **disadvantage(s)** of being physically active? |
| What kind of physical activities do you usually do during a normal day? | - With whom? Where? Organised? - What is your definition of being physically active? |
| Do you think you do sufficient physical activity? |  |
| Would you like to be more physically active? | - How would you do that? - Have you ever tried that? How did you try and how did you experience that? - What makes it **difficult** for you to be physically active? - What makes it **easier** for you to be physically active? |
| By whom are you **supported** to be physically active? | - Which people **discourage** you from being physically active (disapprove of you being physically active)? - How do you experience that? |
| Do you talk with others about physical activity behaviour? About what? | - From whom do you take advice? How do you experience that advice? - What are for you trustworthy sources regarding information about sufficient physical activity? |
| Would you like to receive guidance to be (more) physically active? | - From whom? - About what topic would you like to receive advice? |
| Where and when would you like to receive guidance for physical activity? | - Individually or in a group? |
| What kind of physical activities would you prefer? |  |
| Where would you like to be physically active? | - Indoors or outdoors? - Sports club? - With whom? |
| ***Lifestyle (guidance)*** |  |
| So far, we mainly talked about eating healthily and being physically active. What else is important for you for a healthy life? | - Are those things more important than eating healthily or doing sufficient physical activity? |
| How could we help/stimulate you to live healthily? | - If we offered you guidance, what should that guidance look like? - What is important to you regarding (lifestyle) guidance? |
| ***Additional topics, if there is enough time:***   - ***Smoking*** - ***Participating in research*** |  |
